# Auditory Streaming as an Online Classification Process with Evidence Accumulation

**DOI:** 10.1371/journal.pone.0144788

**Published:** 2015-12-15

**Authors:** Dana Barniv, Israel Nelken

**Affiliations:** 1 Edmond and Lily Safra Center for Brain Sciences, Hebrew University, Jerusalem, Israel; 2 Department of Neurobiology, Silberman Institute of Life Sciences, Hebrew University, Jerusalem, Israel; UNLV, UNITED STATES

## Abstract

When human subjects hear a sequence of two alternating pure tones, they often perceive it in one of two ways: as one integrated sequence (a single "stream" consisting of the two tones), or as two segregated sequences, one sequence of low tones perceived separately from another sequence of high tones (two "streams"). Perception of this stimulus is thus bistable. Moreover, subjects report on-going switching between the two percepts: unless the frequency separation is large, initial perception tends to be of integration, followed by toggling between integration and segregation phases. The process of stream formation is loosely named “auditory streaming”. Auditory streaming is believed to be a manifestation of human ability to analyze an auditory scene, i.e. to attribute portions of the incoming sound sequence to distinct sound generating entities. Previous studies suggested that the durations of the successive integration and segregation phases are statistically independent. This independence plays an important role in current models of bistability. Contrary to this, we show here, by analyzing a large set of data, that subsequent phase durations are positively correlated. To account together for bistability and positive correlation between subsequent durations, we suggest that streaming is a consequence of an evidence accumulation process. Evidence for segregation is accumulated during the integration phase and vice versa; a switch to the opposite percept occurs stochastically based on this evidence. During a long phase, a large amount of evidence for the opposite percept is accumulated, resulting in a long subsequent phase. In contrast, a short phase is followed by another short phase. We implement these concepts using a probabilistic model that shows both bistability and correlations similar to those observed experimentally.

## Introduction

When a listener is exposed to an auditory scene, she is able to segregate it into different sound entities even when the sounds generated by the different sound entities are interleaved in time. The temporally-continuous perceptual representation of a single auditory entity was termed a “stream of sound” [1]. A common paradigm for studying auditory streams consists of presenting sequences of two alternating pure tones to subjects and registering their reported perception [2]. The sequences are presented as “ABAB…” or “ABA-ABA-…“, where the dash represents a silent interval. Subjects usually report one of two perceptions: either all tones are perceived as parts of a single entity (corresponding to one stream percept), or the A tones are perceived separately from the B tones (corresponding to two streams percept). Listeners report on-going switching between the two percepts, without settling into a single, stable percept, where each phase (integration or segregation) persists for several seconds [3]. The phenomenon is therefore bistable. The frequency separation between the high and the low tones has a considerable impact on perception: for intermediate frequency separation, the initial percept tends to be integration, and the proportion segregation reports increases with time. This process is often referred to as “the build-up of streaming”. The final, stationary, probability of segregation depends on stimulus parameters: the larger the frequency separation between the tones, or the faster the sequence, the stronger the tendency for segregation [4]. Extreme values of frequency separation do not yield build-up, as demonstrated by Deike et al [5]: large frequency separations lead to segregation already at the beginning of the sequence, while small frequency separations start with an integration percept and do not segregate later on.

A previous study found that phase durations in auditory streaming are statistically independent [3]. While correlations in auditory streaming have not been reported, there are some reports of positive correlations between phase durations in two studies of binocular rivalry [6,7], a visual bistable phenomenon with spontaneous transitions between two percepts, that is often compared to auditory streaming. These correlations were reported for successive phase durations of the same type (separated by one phase of the opposite percept). The major experimental result of the current paper is the demonstration that successive phase durations in auditory streaming do show significant positive correlations.

A computational model of streaming should therefore account for several properties: the build-up of streaming, the bistability expressed in back and forth switching between percepts, and the positive correlations between successive phase durations. Most theoretical accounts of streaming do not provide explanation for this ensemble of properties. A number of models consider segregation to be related to channeling, i.e. separate neuronal representations of different streams [8–10], possibly explaining segregation of alternating sequences, but not bistability. Taking this hypothesis further, Fishman et al [11] offered the population-segregation model, where segregation occurs when the two stimuli are represented by mostly separate neuronal populations; build-up occurs in this model because of the ubiquitous adaptation of neuronal responses during the presentation of a long sequence of sounds. This hypothesis was quantitatively tested by comparing predictions based on the time course of the responses of neurons in macaque auditory cortex, as well as in guinea pig cochlear nucleus, to the dynamics of build-up of streaming measured in humans [12,13]. While the Fishman model can in principle be extended to account for bistability as well, such extension would require neuronal responses to switch between segregated and integrated representations with the appropriate long time scales; no evidence has been found for such dynamics in the neuronal responses. Extensions of the Fishman model such as a temporal coherence model [14], as well as neural oscillator models [15,16], account for the build-up of streaming but lack mechanisms for on-going switching between integration and segregation. A model by Mill et al [17], where sound entities are identified as predictable patterns, accounts for bistability in response to periodic patterns, and may have correlation between successive phase durations, induced by neural adaptation with long time constants. However, it is tailored for periodic patterns; natural stimuli are usually non periodic (although the model of Mill et al. [17] may be extended to deal with non periodic patterns). The approach we will suggest here does not require the input to be periodic.

The dynamics of bistable phenomena in the visual modality, in particular binocular rivalry, is frequently modeled by neuronal competition models, using adaptation or noise [18] to drive switching. We argue that this family of models is not appropriate for auditory streaming. Whereas in binocular rivalry, switching occurs between two qualitatively similar states (the two images presented to the left and right eyes), in auditory streaming the two alternatives consist of qualitatively different interpretations of the entire scene, which contain a different number of streams. When modeling binocular rivalry, the association of each percept type with the activity of a distinct neuronal population [19–21] is a reasonable assumption since a percept corresponds to a single image. In contrast, it is less natural to model segregation phases of auditory streaming as the activity of a single neuronal population, since this percept corresponds to the co-existence of two streams. It would be much more reasonable to represent each stream, rather than each percept, by a distinct neuronal population; in such a representation, perceptual switching will correspond to toggling between activity in a single population and activity in both.

In this study, we propose a new framework for interpreting auditory scenes, and use a concrete implementation of this framework to account jointly and naturally for bistability and positive correlations between successive phase durations, as well as for the build-up of streaming at intermediate frequency differences.

## Results

### Subsequent Phases of Segregation and Integration are Positively Correlated

We analyzed a large data set of long sequences of perceptual decisions in response to “ABA-” sequences, collected by Hupé, Joffo and Pressnitzer [22] and provided to us courtesy of D. Pressnitzer (Data Set I). There were 16 subjects, each underwent 6 trials of 4 minutes of the ABA- stimulus, with frequency difference of 5 semitones between A and B, and onset to onset intervals of 120 ms or 240 ms (see Methodsfor details). Across all trials and subjects, the average phase duration was 7.8±4.1 s (mean±std) for integration and 8.4±6.0 s for segregation, and the fraction of time spent in segregation was 0.51±0.13. The first and last phases were excluded from each trial.

**Fig 1:**
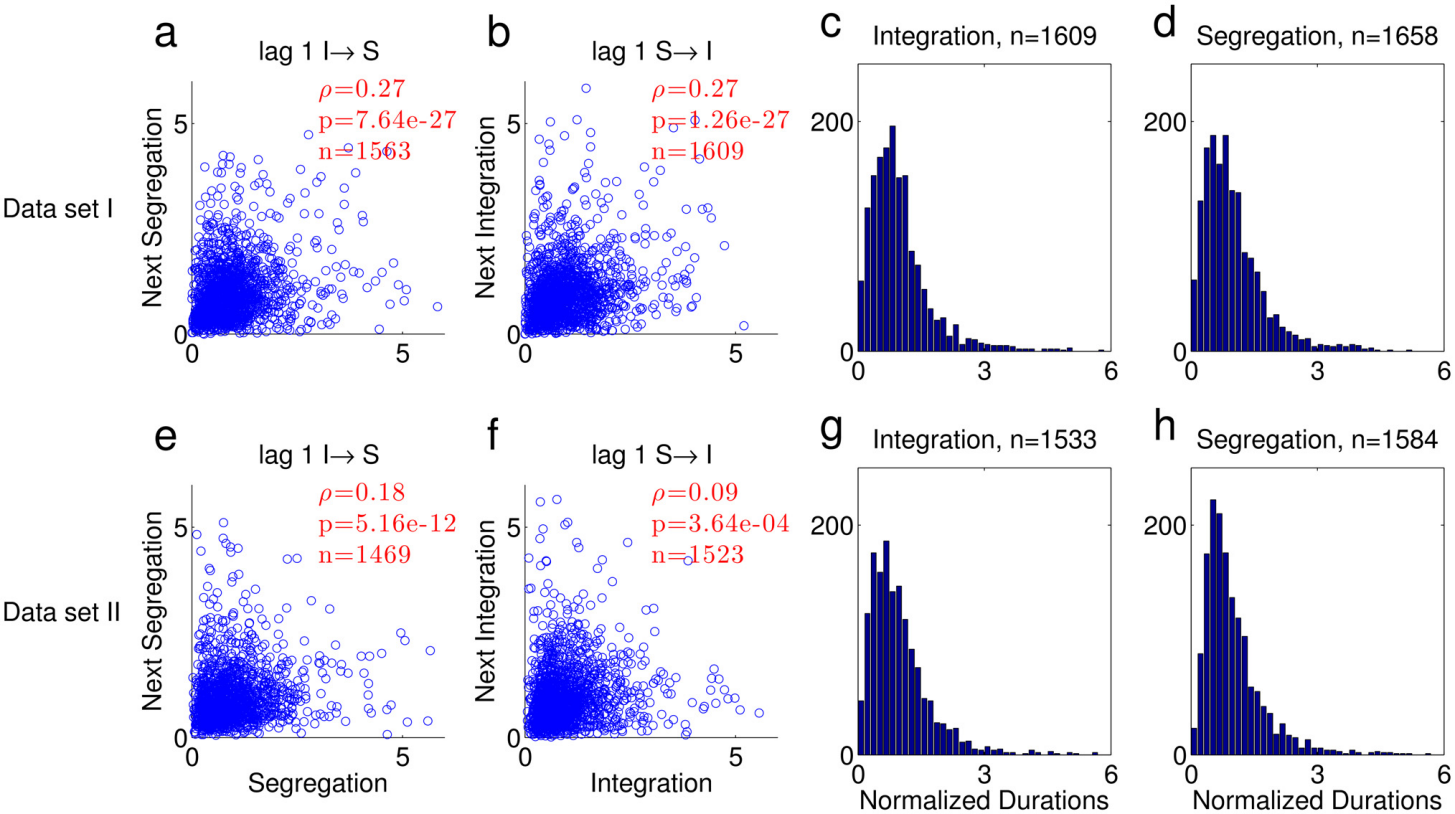
Phase duration analysis. (a-d) Data Set I (9). (a-b) Scatter plots of normalized durations. The correlation coefficient between phase durations for each scatter is indicated in red with the corresponding p value and number of pairs (ρ, p and n respectively). (a) Segregation following integration. (b) Integration following segregation. (c-d) Histograms of normalized phase durations. The number of phases is indicated in the title. (c) Integration. (d) Segregation. (e-h) The same analysis for Data Set II.

Fig 1a and 1b plots the normalized duration of each phase against the duration of the next, for the two possible transitions. Importantly, durations were normalized to the average value of each percept type separately within each trial and subject, in order to avoid spurious correlations due to inter-subject and inter-trial differences in switching behavior (see Methods). For subsequent phase durations (lag 1), the correlation was significantly larger than 0. The correlations between phase durations that were one or two phases apart (lag 2, lag 3), were not significantly different from 0 (lag 2: I→I ρ = 0.049, p = 0.058, n = 1515, S→S ρ = 0.051, p = 0.045, n = 1563; lag 3: I→S ρ = 0.015, p = 0.557, n = 1469, S→I ρ = -0.053, p = 0.038, n = 1515; throughout the paper, we consider p<0.01 as statistically significant). These results do not depend on the details of the analysis. Similar correlations were found when excluding trials that had less than 10 perceptual switches (see Part A in S1 Appendix). The distribution of phase durations is shown in Fig 1c and 1d.

We replicated these results using a similar experimental design (details in Methods) to collect additional data (Data Set II, see S1 Dataset). There were 21 subjects, each undergoing 6 trials of 4 minutes of the ABA- stimulus, with an interval of 5 semitones between A and B, and onset to onset interval of 110 ms. Across all trials and subjects, the average phase duration was 6.0±7.1 sec for integration and 8.1±8.6 sec for segregation, and the fraction of time spent in segregation was 0.59±0.12. Correlations between subsequent durations were smaller compared to Data Set I, but still highly significant, as shown in Fig 1e and 1f. As in Data Set I, lag 2 and lag 3 correlations were not significantly different from 0 (lag 2: I→I ρ = 0.013, p = 0.635, n = 1410, S→S ρ = 0.049, p = 0.064, n = 1460; lag 3: I→S ρ = 0.008, p = 0.777, n = 1348, S→I ρ = -0.025, p = 0.357, n = 1401). As in Data Set I, similar correlation values were found when excluding trials that had less than 10 perceptual switches (see Part A in S1 Appendix).

**Fig 2:**
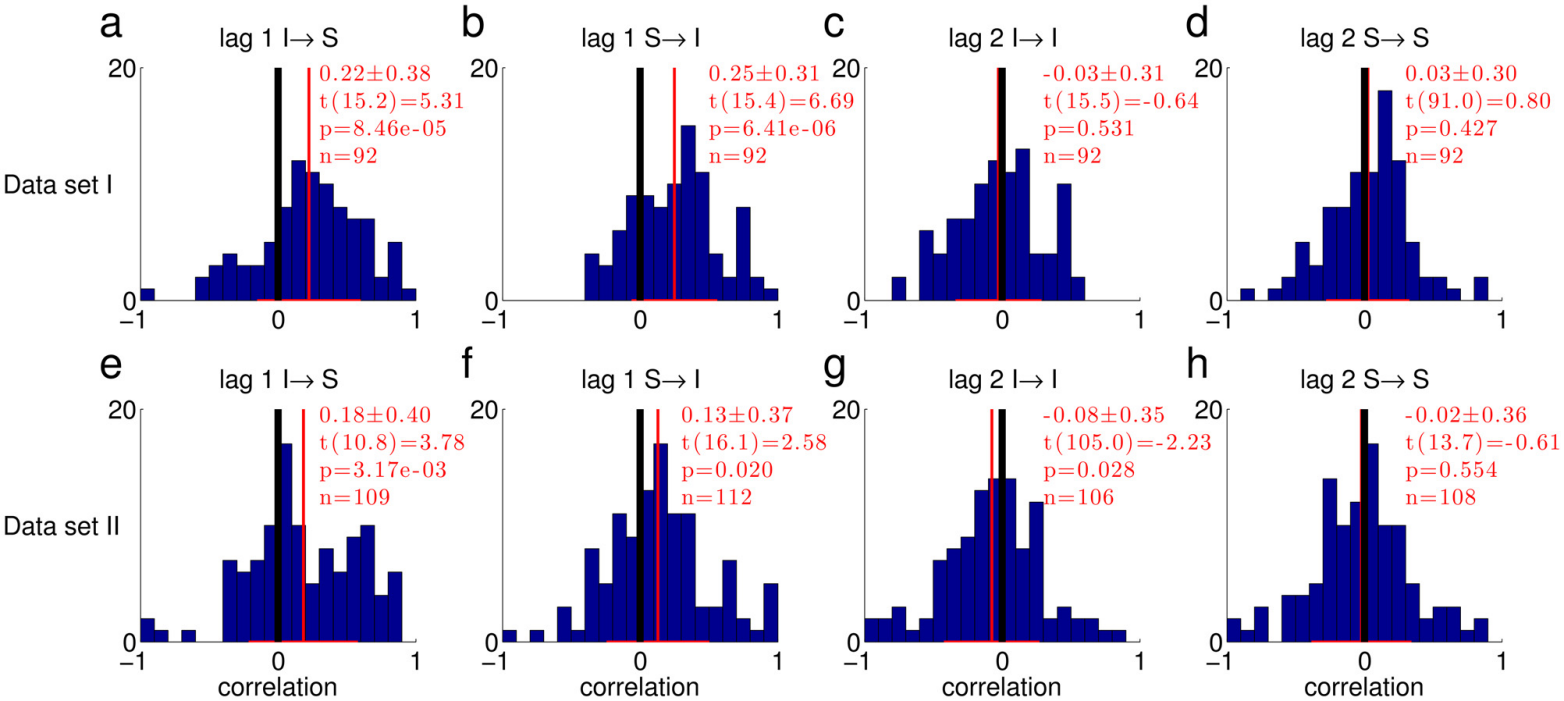
Histograms of correlation coefficients between phase durations in single trials. The transition types are marked above each histogram (for example, I→S denotes transitions from integration to segregation). The mean and standard deviation are indicated in red in each panel, followed by the t-statistic of the fixed effect, its significance and the number of trials for which it was possible to calculate the correlation. The black vertical lines mark zero correlation, and the red vertical lines mark the mean of each distribution. (a-d) Data Set I. (e-h) Data Set II.

When combining data from different subjects and conditions, spurious positive correlation may be produced, for example when the average phase duration differs between subjects/conditions. Although the data in Fig 1 was normalized, the normalization may not be reliable enough, giving rise to the observed correlations. To show that the correlation in successive phase durations was not due to such effects, we also calculated correlation coefficients separately for each switch type in each individual trial. Fig 2 shows a histogram of the single trial correlations between subsequent phases (lag 1, integration followed by segregation and vice versa) and between phase durations that are one phase apart (lag 2, two subsequent integration or segregation phases) for both data sets. Using linear mixed effects analysis [23] with subject as a random factor, we found a significant deviation of the histogram towards positive correlation values for lag 1 in Data Set I (Fig 2a and 2b; see Methodsfor details), and for the I→S transition in Data Set II (Fig 2e). For both Data sets, lag 2 correlations were not significantly different from 0 (Fig 2c, 2d, 2g and 2h), and lag 3 correlations as well (Data Set I: lag 3 I→S t(14.4) = 0.341, p = 0.738, S→I t(91) = -1.603, p = 0.112. Data Set II: lag 3 I→S t(102) = 1.033, p = 0.304, S→I t(16.7) = -1.579, p = 0.133). The random subject effect did not reach significance for any switch type in any data set, except lag 1 S→I in Data Set II (Data Set I: lag 1: I→S p = 0.6, S→I p = 0.4; lag 2: I→I p = 0.09, S→S p = 1; lag3: I→S p = 0.08, S→I p = 1. Data Set II: lag 1: I→S p = 0.3, S→I p = 0.01; lag 2: I→I p = 1, S→S p = 0.9; lag3: I→S p = 1, S→I p = 1). To summarize, for Data Set I, lag 1 correlation was significantly larger than 0, and for Data Set II, one of the two types of lag 1 correlation was significantly larger than 0, while the second type was borderline significant, and seemed to be affected by the subject random factor. In both data sets, lag 2,3 correlations were not significantly different from 0.

In a previous study, Pressnitzer et al. [3] reported that subsequent phase durations are uncorrelated. They analyzed 23 trials of 4 minutes, a data set which is much smaller than those we analyzed here (96 trials in Data Set I; 126 trials in Data Set II). We recalculated the correlations of this data set [3], which was also provided to us courtesy of D. Pressnitzer, with the same approach used for Data Sets I and II. While the lag 1 I→S correlation was null (ρ = -0.065, p = 0.339, n = 219), the lag 2 S→I correlation was similar in size to that found in the other data sets, but didn't reach our strict criterion for significance (p<0.01) because of the relatively small amount of data (ρ = 0.151, p = 0.026, n = 217). Lag 2 and lag 3 correlations were also non-significant (p>0.2). Thus, the null correlations reported by Pressnitzer et al. [3] may be attributed, at least partially, to the smaller size of the data set, which reduced the power of the statistical tests.

To summarize, two independently collected large data sets exhibit significant positive correlation between subsequent phase durations. Correlations between phases that are one or two phases apart were small or null. In the rest of the paper, we will use this correlation structure as an important constraint on models of streaming.

### Streaming as a process of evidence accumulation

We suggest that streaming is a consequence of an evidence accumulation process over time. The notion of evidence we use here is akin to probability—the evidence for integration is the complement of the evidence for segregation. Intuitively, the more evidence for the opposite percept, the higher the probability that a perceptual switch would occur. During an integration phase, evidence for segregation accumulates; during a segregation phase, evidence for integration accumulates (or equivalently, evidence for segregation decreases). The amount of evidence is continuous across switches, so that in order for the opposite switch to occur next, the amount of evidence for the other precept has to increase sufficiently.

Regardless of implementation details, this framework yields positive correlation between durations of successive phases: during a long phase, a large amount of evidence for the opposite percept accumulates, resulting in a typically long subsequent phase, while a short phase would be followed by another short phase. In order to demonstrate that this framework is reasonable for streaming, we propose next a specific implementation that also satisfies the other experimental constraints: the effect of frequency separation and presentation rate on the probability of segregation.

### An Implementation of the Evidence Accumulation Process as an Online Classification Process

In the model described here, classes correspond to streams, and when an input is classified to a certain class, it is declared to be part of the corresponding stream. Classification and update of class parameters are performed using a straightforward set of probabilistic rules, which produce competition between integration (all inputs are classified to the same stream) and segregation (inputs are classified to several classes depending on their identities).

#### The Classification Algorithm

We assume that the auditory input undergoes a preprocessing stage, where it is segmented into time discrete elements (corresponding to syllables in speech, notes in music, or tones in an ABA- sequence). These are then represented as a vector in some d-dimensional feature space.

The sequence of incoming elements is fed into the classification process, where each element is attributed (classified) to one of the classes. A class corresponds to a stream, and all the elements that are assigned to that class belong to the same stream. When all inputs are classified to one class, only one stream is perceived. When some of the inputs are classified to one class and other inputs to another, the sequence is perceived as segregated, and the two classes represent the two streams. The assignment of elements to streams is determined by the classification results. In the special case of the alternating tone sequences used to study auditory streaming, classification of both A’s and B’s to the same class is interpreted as integration, while classification of A’s to one class and B’s to another is interpreted as segregation. It is important to note here that classifying two elements to the same class does not imply that they are perceptually indistinguishable, but rather that they belong to the same stream.

The classification process assumes that each element x of the sequence is drawn from a mixture distribution with n components, representing n different classes:

$$p\left(x\right)=\sum_{k=1}^{n}\pi_{k}p\left(x|c=k\right)$$

Where *c* ∈ {1,2,…,*n*} is the class that generated x,*π*_*k*_ is the a-priori probability of class k to occur (referred to as the mixing probability), and *p*(*x*|*c* = *k*) is the conditional likelihood of x given that it was generated by class k. For simplicity we assume that the inputs are represented by a single feature, for example tone frequency for streaming experiments. The feature space is therefore 1-dimensional, and so is the conditional likelihood. More generally, the same scheme is applicable in any dimensionality, by representing inputs as vectors and distributions as multi-dimensional.

For simplicity, we assume in all the calculations below that the conditional likelihoods are characterized by a small number of parameters. To allow freedom in the shape of the conditional likelihoods, we continue here with generalized Gaussian mixtures, which are described by the set of parameters ${\left\{\pi_{k},\mu_{k},\sigma_{k}\right\}}_{k=1}^{n}$ where $\overset{n}{\underset{k\;=\;1}{\sum }}\pi_{k}=1$, along with a shape parameter β, which is determined a-priori (see Methods). Further simplifying, the parameters ${\left\{\sigma_{k}\right\}}_{k=1}^{n}$ are assumed to be known and equal for all classes, *σ*_*k*_ = *σ*. However, the general approach is valid for other parameterizations of the conditional likelihoods.

Upon arrival of an input element x, it is classified to a class k*. We use here two flavors of the classification process. In one implementation, classification is deterministic: k* = *argmax*_*k*_ {*p*(*c* = *k*|*x*)}, i.e.k* is the class that has the maximal a-posteriori distribution for input x, among all existing classes. Deterministic classification simplifies the analysis of model behavior. In the second, more general type of classification, k* is drawn stochastically from the a-posteriori distribution ${\left\{p\left(c=k|x\right)\right\}}_{k=1}^{n}$. Such stochastic classification may better reflect actual behavior.

Next, the parameters of the mixture are updated with the goal of faithfully representing the statistics of the input sequence, given the arrival of the current input element. Updating the mixture parameters is done as follows:

1. The centroid of the selected class, *μ*_k*_, is shifted towards x, by an amount that is proportional to the **a-posteriori probability** of the class given the input, and to the distance between the centroid and the input:
  $$\mu_{k^{*}}\leftarrow \mu_{k^{*}}+\gamma \;p\left(c=k^{*}|x\right)\left(x-\mu_{k^{*}}\right)$$ (1)
  Where *γ* is an update rate parameter.
2. The mixing probabilities ${\left\{\pi_{k}\right\}}_{k=1}^{n}$ are updated using a different criterion: the increase in the mixing probability of class *k*, after proper normalization (to ensure that the sum of the mixing probabilities remains 1), is proportional to the **conditional likelihood** of the new input *p*(*x*|*c* = *k*), such that the mixing probability of the class with maximum conditional likelihood increases at the expense of the other classes. The change is applied to all mixing probabilities:
  $$\pi_{k}\leftarrow \pi_{k}\left[1+\eta \left(p\left(x|c=k\right)-p\left(x\right)\right)\right]$$ (2)
  The mixing probability increases for classes that are likely to have generated x. The rule therefore gives preference to classes with high conditional likelihood, rather than to classes with high a-posteriori probability (like in the *μ*_*k*_ update).
3. When the likelihood of the incoming element is smaller than some threshold, *p*(*x*)< *θ*, a new class is generated, centered around the element, and assigned a small initial mixing probability *p*_*init*_:

$$\mu_{n+1}\leftarrow x$$

$$\pi_{n+1}\leftarrow p_{init}$$

$$\pi_{k}\leftarrow \pi_{k}\left(1-p_{init}\right)\;\;\;\;\;\;\;\;\;k=1,\ldots ,n$$

n←n+1

**Fig 3:**
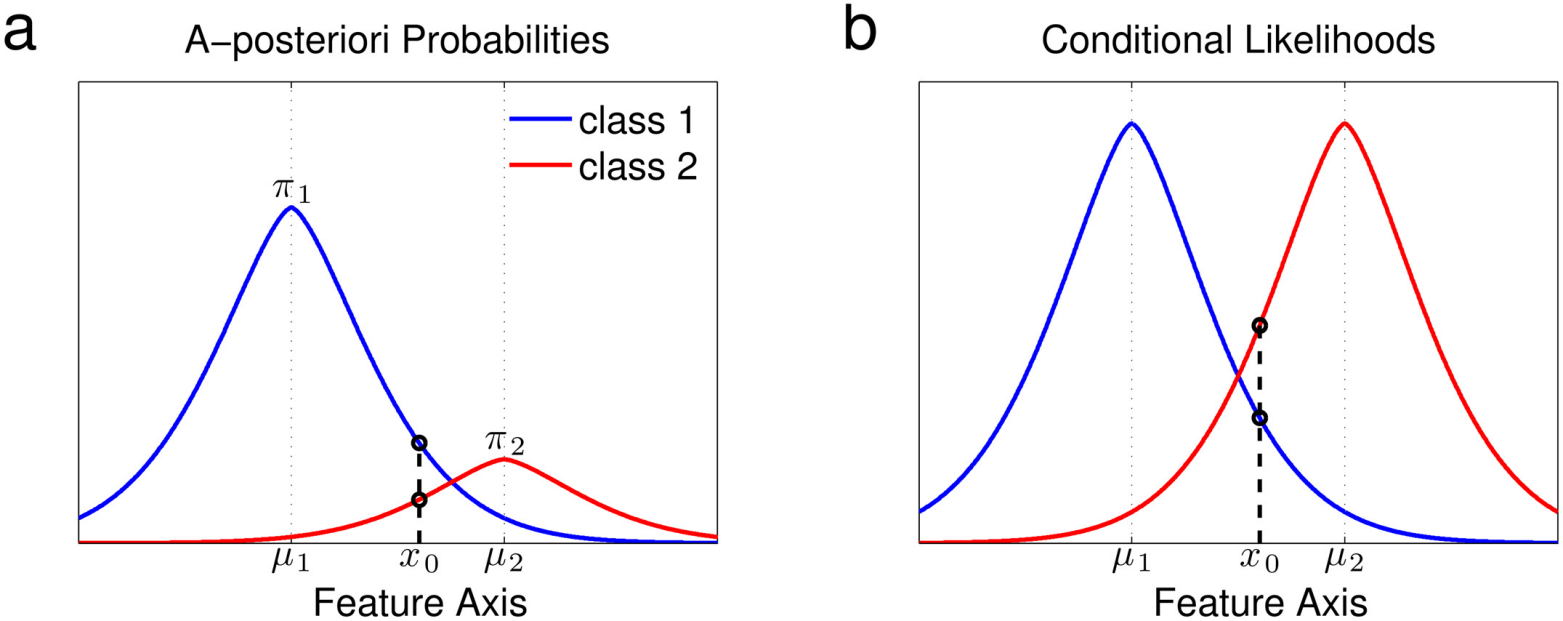
The criteria used for classification and update. (a) The a-posteriori probabilities ***p***(***c*** = ***k***|***c***) for ***k*** = **1, 2**. For the indicated input ***x***_0_, the maximum is obtained in class 1, making ***x*_0_** more likely to be classified to class 1, in which case ***μ***_1_ moves towards ***x*_0_**. (b) The conditional likelihood ***p***(***x***|***c*** = ***k***). For the input ***x*_0_**, the maximum is obtained in class 2, resulting in an increase in the mixing probability ***π***_2_.

The difference between updates based on the a-posterior probability and updates based on the conditional likelihood is crucial for the behavior of the model, since it produces competition between the integration and segregation interpretations. To illustrate this difference, consider the example in Fig 3. Two classes are illustrated, with the mixing probability *π*_1_ of class 1 substantially larger than that of class 2. For the indicated input *x*_0_, the a-posteriori probability, used for classification and centroid update, is maximal for class 1 (Fig 3a); in contrast, the maximum conditional likelihood, used for mixing probability update, is obtained for class 2 (Fig 3b). *x*_0_ is therefore more likely to be classified to class 1, in which case *μ*_1_ will move towards *x*_0_. Regardless of this classification decision,*π*_2_ will increase, whereas *π*_1_ will decrease. Thus, in the probable case where *x*_0_ is classified to class 1, *p*(*x*|*c* = 1) increases but *π*_1_ decreases. The product of the two terms, which is proportional to the a-posteriori probability *P*(c = 1|*x*_0_) ∝ *P*(*x*_0_|*c* = 1)*π*_1_, may therefore increase or decrease depending on the current mixture parameters and the update rate parameters. If the decrease in *π*_1_ dominates, then inputs similar to *x*_0_ that arrive later in the sequence will become more likely to be classified to class 2. On the other hand, if the change in *μ*_1_ dominates, such inputs will be more likely to be classified to class 1. We therefore get accumulated evidence for a new interpretation of the scene, competing with the tendency to adhere to the current scene interpretation: the former is mathematically expressed in the mixing probabilities update, while the latter is expressed as the centroid update of the selected class.

To understand the competition that this algorithm generates, let us consider an example scenario. Assume all incoming inputs are concentrated in the same region in the feature space, and are classified to the same class. Assume now that an input from a different region arrives, such that its likelihood is lower than the threshold. A new class will be created around it, but the input will probably not be classified to it, due to the low initial mixing probability of this class. The new class does not take part in classification, but its existence is an indicator of the possibility that a new stream of sound may be occurring. If no more inputs in the vicinity of the new input arrive, the mixing probability of the new class will decrease and remain of no importance for classification. However, if more inputs in the same region arrive, the mixing probability of the new class may gradually increase, possibly up to the point when inputs similar to the new input begin to be classified to it.

The mixing probability of the new class measures the amount of evidence accumulated for the existence of a new stream. As long as none of the elements are classified to the new class, the corresponding new stream of sound is perceptually irrelevant. The rate of evidence accumulation for the existence of this stream (instantiated in changes in its mixing probability), in conjunction with the rate of the process that tends to integrate the two classes (namely the rate of updating *μ*_k*_ towards the new stimulus), determines if and when this new stream will be unveiled, corresponding to a switch in perceptual organization.

#### Generating Switching Behavior

We first examine behavior of the algorithm for the case where classification is deterministic, i.e. k* = *argmax*_*k*_ {*p*(*c* = *k*|*x*)}, and show that the algorithm produces periodic switching between integration and segregation.

**Fig 4:**
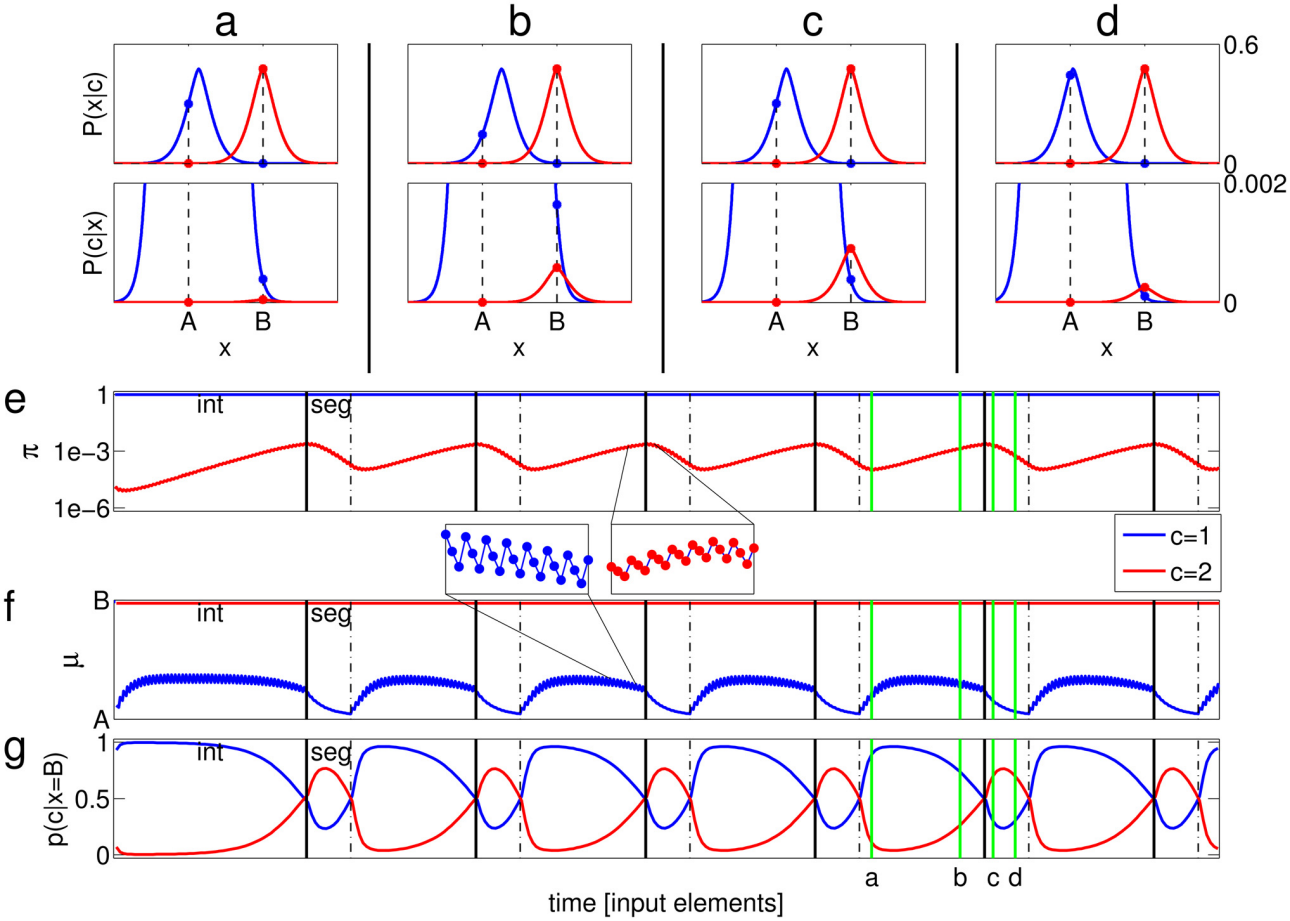
State of the mixture distribution in response to an ABA- sequence, shown at several time points. The top plot in each panel shows the conditional likelihood of the two classes, ***P*(*x*|*c* = *k*)** as a function of ***x***, and the bottom shows the a-posteriori probability ***P*(*c* = *k*|*x*)**, with the ordinate magnified so as to observe the relationship between ***P*(*c* = 2|*B*)** (red dot above B) and ***P*(*c* = 1|*B*)** (blue dot above B). (a) During integration (where both inputs are classified to class 1), ***μ*_1_** approaches a value midway between A and B, causing a decrease in ***P*(*A*|*c* = 1)**. (b) This decrease causes ***π*_2_** to undergo a net increase. (c) The increase in ***π*_2_** results in a switch to segregation: B is now classified to class 2. (d) ***μ*_1_** returns towards A; the result is a net decrease in ***π*_2_**. (e-g) The dynamical variables as a function of time. Black vertical lines indicate switches from integration to segregation (solid) and vice versa (dashed), as indicated by the labels “int” and “seg” in the first two phases. Green vertical lines indicate the time points sampled at (a-d). (e) ***π*_1_, *π*_2_**, plotted in log scale. The inset illustrates the fast oscillations of ***π*_2_**, where each point indicates one time step; notice there are two down steps after each up step, because A occurs twice after every occurrence of B in the ABA- sequence. (f) ***μ*_1_, *μ*_2_**. The inset illustrates the fast oscillations of ***μ*_1_**. (g) The value of ***P*(*c* = 1|*B*)** and ***P*(*c* = 2|*B*)**, which sum up to 1. In this simulation ***γ* = 0.1, *η* = 0.5**, the stimulus elements are **Δ = 5** apart, and the shape of the conditional likelihood is generalized normal with **β = 1.5** and a variance of 1.

Fig 4 illustrates the results of the classification algorithm for deterministic classification of the ABA- sequence. Elements A and B are represented as points on the feature axis. In its initial state, the mixture has two classes, centered close to A and B. Class 1 has a much larger mixing probability, such that both A and B are classified to it, and the percept is of integration (Fig 4a, lower panel).

The structure of the alternating stimulus results in fast fluctuations of the mixture parameters values: at every presentation of A, *μ*_1_ moves towards A and *π*_2_ decreases, and at every presentation of B, *μ*_1_ moves towards B and *π*_2_ increases (see inset in Fig 4e). These fluctuations ‘ride’ on a slower trend, whose behavior depends on the details of the update rules, as well as on the parameters of the model and the stimulus. In our case, since *π*_1_ is almost 1, *μ*_1_ effectively moves towards B (Eq 1, Fig 4b, upper panel; note that class 1, in blue, is no longer centered close to A). As a result, the conditional likelihood *P*(*A*|*c* = 1) becomes substantially smaller than *P*(*B*|*c* = 2), and the increase in *π*_2_ upon presentation of B becomes more significant than the decrease in *π*_2_ upon presentation of A (Eq 2), giving rise to a net increase in *π*_2_. Eventually *P*(*c* = 2|*B*) exceeds *P*(*c* = 1|*B*) (Fig 4c, lower panel), and the result is classification of B to class 2, representing a switch to segregation. From that point, *μ*_1_ begins to return towards A, yielding a net decrease in *π*_2_ (Fig 4d). The mixture slowly returns to the integration state of Fig 4a, where B is classified to class 1.

In this simulation of the ABA- sequence, the process converged to periodic switching between integration and segregation, following a transient period that depended on initial conditions. The periodicity is evident from Fig 4e and 4f, where the dynamics of *π*_1,2_ and *μ*_1,2_ are displayed. Notice that since the center of each class is updated according to the inputs classified to that class, and since only B’s are ever classified to class 2, *μ*_2_ = *B* throughout the simulation. In addition,*π*_1_ is equal to 1 −*π*_2_, and is therefore close to 1 all the time. As mentioned, switches between phases occur when *P*(*c* = 1|*B*) = *P*(*c* = 2|*B*), namely when they are both equal to 0.5, as shown in Fig 4g. Our analysis shows that periodic behavior is obtained for a wide range of simulation parameters, input properties and initial conditions.

Similar results were obtained for the ABAB sequence (not shown). The main difference is that the two classes are now symmetrical, so two states of integration occur—when both stimuli are assigned to class 1 and when both are assigned to class 2. We note that for both ABA- and ABAB there is inherent asymmetry between the model behavior during integration and segregation: during integration inputs are classified to one class, and during segregation to two classes, so that the update process is essentially different. Therefore, we do not expect the typical durations of the integration and segregation phases to be similar to each other.

**Fig 5:**
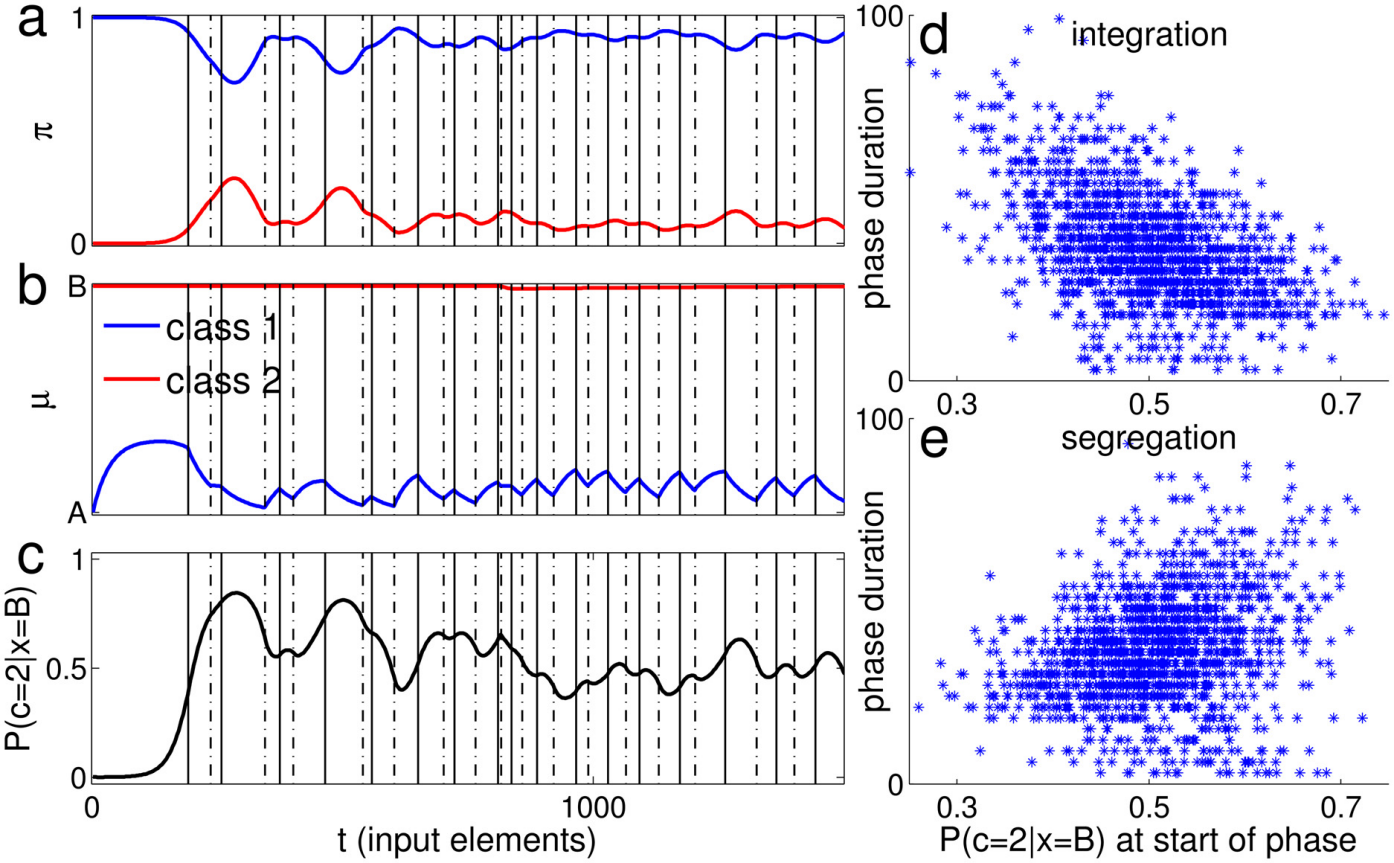
The stochastic model. (a) π as a function of time. (b) μ as a function of time. (c) The probability of B being classified to class 2, ***P*(*c* = 2|*B*)**, determining the probability of segregation, since ***P*(*c* = 2|A) ≈ 0**. For clarity, in all panels, only the values after arrival of input B are shown, namely one value every three time steps. Black vertical lines indicate switches from integration to segregation (solid) and vice versa (dashed). (d-e) Phase durations are plotted against the value of ***P*(*c* = 2|*B*)** at the start of the phase, separately (d) for integration and (e) for segregation. In this simulation ***γ* = 0.03, *η* = 0.1, *K* = 6**, the stimulus elements were **Δ = 0.6** apart, and the shape of the conditional likelihood was generalized normal with **β = 0.5** and a variance of 1. The mixture was initialized with two classes centered at A and B, and ***π*_2_** was initially 0.0001. The phases are all drawn from a single long simulation run.

**Fig 6:**
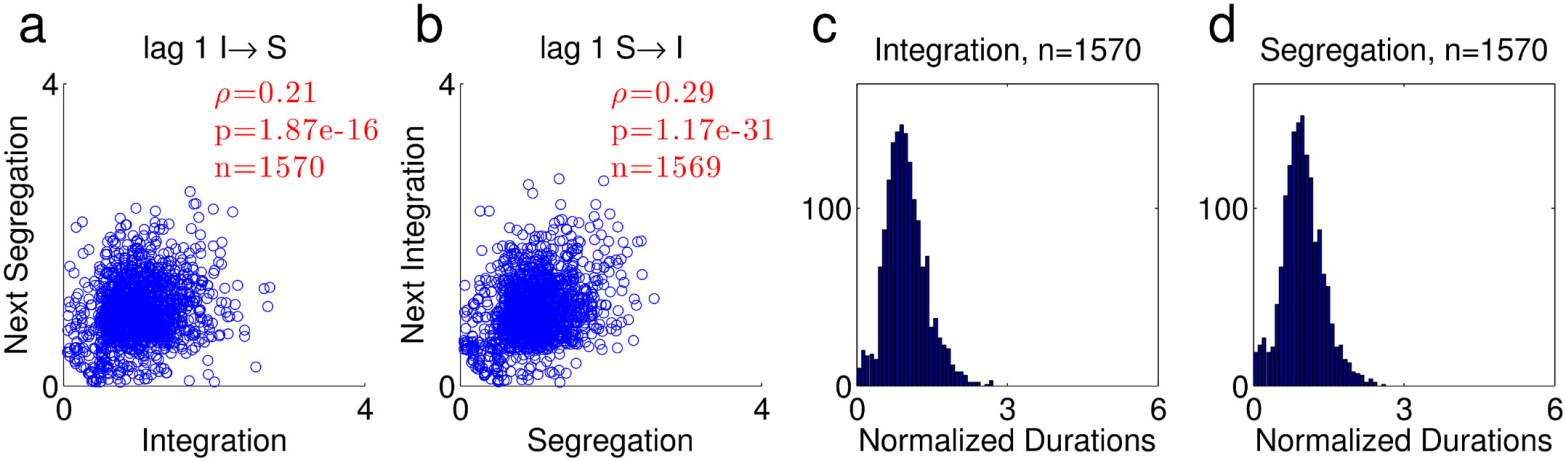
Analysis of phase durations from the same simulation as in Fig 5. (a-b) Scatter plots of normalized durations. The correlation coefficient between phase durations for each scatter is indicated in red along with its p-value and number of couples (ρ, p and n respectively). (a) Segregation following integration. (b) Integration following segregation. (c-d) Histograms of normalized phase durations. The number of phases is indicated in the title. (c) Integration. (d) Segregation. The mean phase durations were 36.1 input elements for integration and 36.2 for segregation.

Periodic switching between integration and segregation is obviously not in line with the highly variable phase durations measured in experiments. Since the model is probabilistic, it is only natural to introduce stochasticity by considering probabilistic decisions based on the distributions ${\left\{p\left(c=k|x\right)\right\}}_{k=1}^{n}$ maintained in memory. For the ABA- sequence, A is almost exclusively classified to class 1 since *p*(*c* = 1|*A*) >> *p*(*c* = 2|*A*), as evident in Fig 4a–4d (lower panels). Therefore, evidence accumulation is well represented by the probability *p*(*c* = 2|*B*): during integration, the increase in this value represents evidence for segregation, and during segregation, the decrease in this value represents evidence for integration. Perceptual switches should therefore take place stochastically, biased by the value of *p*(*c* = 2|*B*). To implement this in the model, when *K* inputs of type B have been classified to class 2 during integration, a switch to segregation occurs, and vice versa—when *K* B’s have been classified to class 1 during segregation, a switch to integration occurs. The resulting behavior of the dynamic variables and switching times is shown in Fig 5. A reasonable scheme would set some allowable time scale for this counting process; nevertheless, adding such time limitation and fitting its value to experimental durations will most likely not change our results qualitatively, since the phase durations in our simulations tend to be short (see Fig 6c and 6d).

As predicted, during a long integration phase, a large amount of evidence for segregation accumulates and is expressed as a large value of *p*(*c* = 2|*B*), yielding a long subsequent segregation phase; a short integration phase provides time for only moderate evidence accumulation for segregation, so that the value of *p*(*c* = 2|*B*) tends to be intermediate at the end of the phase, and the next phase is typically shorter. A similar evidence accumulation for integration occurs during segregation phases, leading to the opposite dependence on *p*(*c* = 2|*B*). In Fig 5d and 5e we show the correlation between the value of *p*(*c* = 2|*B*) at the start of the phase, and the phase duration: for integration ρ = -0.49, p = 1.12e-96 and for segregation ρ = 0.25, p = 1.27e-23 (the number of sampled phases was 1572, similar to the number of phases in the data, see Fig 1). The value of *p*(*c* = 2|*B*) indeed tends to increase during integration and decrease during segregation (Fig 5c), but this tendency does not occur immediately after the perceptual switch; there is an initial overshoot in the start of the segregation phase as well as an undershoot in the start of the integration phase. This behavior reflects the competition discussed above, where *π*_2_ update opposes the *μ*_1_ update, such that the scene interpretations compete. In the simulation, the change in *π*_2_ finally overcomes the change in *μ*_1_, resulting in termination of the overshoot/undershoot. The existence of the overshoot and undershoot depends on the parameters used in the simulation. For comparison, in the simulation of Fig 4, where the parameters are different, there is undershoot in *p*(*c* = 2|*B*) in the beginning of integration (Fig 4f), but the overshoot in the beginning of segregation is almost non-existent. In addition to the difference in parameters, the simulation of Fig 5 is stochastic, so that phases may terminate at different stages of the competition, resulting in a variety of overshoot/undershoot sizes.

As a result of this evidence accumulation process, positive correlation exists between durations of subsequent phases, as shown in Fig 6a and 6b (compare to Fig 1a, 1b, 1e and 1f). Lag 2 and 3 correlations were either non-significant or small compared to lag 1 correlation (correlation values from the same simulation of Figs 5 and 6. lag 2: I→I ρ = -0.075, p = 2.79e-3, n = 1569, S→S ρ = -0.0031, p = 0.903, n = 1569; lag 3: I→S ρ = 0.062, p = 1.41e-2, n = 1569, S→I ρ = -0.077, p = 2.19e-3, n = 1568). The histograms of phase durations are similar in shape to those produced from the data, albeit with notable qualitative differences, mainly in the abundance of short durations and lack of long tail in the simulated durations (Fig 6c and 6d; compare to Fig 1c, 1d, 1g and 1h). We emphasize that the algorithm is implemented in its simplest form, providing a conceptual framework rather than an accurate replica of experiments. A fuller model may include additional features to better fit the experimental distribution. The long tail, for example, is typically found in reaction time distributions, and is sometimes related to lapses of attention [24]; the effect of attention is however extraneous to the competition between interpretations that is studied here and therefore was not implemented here.

In the simulation of Figs 5 and 6, the parameters γ and η that govern the dynamical behavior of the model were selected such that lag 1 correlations exist. The parameter K and the stimulus feature Δ were selected such that the mean phase durations were approximately equal and in the range of 24–57 input elements, which fits the range of durations in our experimental results (see below). However, positive correlations at lag 1 were found consistently for a wide range of the parameters. We calculated the correlation coefficient for 900 simulation runs with 0.001≤ *γ* ≤ 0.3, 0.001 ≤ *η* ≤ 0.3. In 813 of the simulation runs, switching occurred, and correlations were calculated. The lag 1 correlation was significantly positive with mean±std 0.159±0.136, range of [-0.34,0.81], and 744/813 positive values for the I→S transition; the same values were 0.495±0.215, [-0.35,0.83], 785/813 for the S→I transition. Lag 2 correlations were substantially smaller (although significantly larger than 0, 0.043±0.123, [-0.27,0.82], 529/813 (I→I) and 0.095±0.171, [-0.25,0.55], 520/813 (S→S)).

The initialization of the mixture distribution variables determines the initial behavior of the algorithm. Assuming an initial state with one predominant class close to A, this predominant class will initially capture both A and B and its centroid will move towards them. Under the appropriate conditions, a new class will form around B with a small mixing probability *π*_n + 1_ = *p*_*init*_. In this case, if *p*_*init*_ is small enough (much smaller than the long-term typical value of *π*_n + 1_), then the initial percept will be of integration; the smaller *p*_*init*_, the longer this initial integration phase. In Fig 5 we set *π*_2_ to be 0.0001 in the beginning the simulation. Since later in the simulation *π*_2_ fluctuates around ~0.1, there was a long initial integration phase that terminated around the time *π*_2_ reached that value.

The time scale of switching may fit perceptual time scales. In the simulation of Fig 6, the average phase duration was around 36 input units, corresponding to 12 ABA- triplets per phase. In Data Set I, the mean phase duration was 7.8–8.4 sec and the presentation rate was 1–2 ABA- triplets per second. In Data Set II, the mean phase duration was 6.1–8.3 sec and the presentation rate was 2.3 ABA- triplets per second. This yields a range of 8–19 ABA- triplets per phase in the data (or 24–57 input elements per phase).

#### The model fulfills the experimental constraints

The fraction of time spent in segregation for the ABA- sequence increases when the frequency separation is larger and when the sequence is faster [4]. In the model, several parameters affect the fraction of time spent in segregation phases. We focus on two parameters: Δ, the distance between the representations of A and B on the feature axis, and σ, the common standard deviation of the conditional likelihoods of all classes. Since σ is assumed fixed in time, the number of parameters can be reduced by defining the normalized difference between stimulus element to be $\tilde{\Delta }=\frac{\Delta }{\sigma }$ and the normalized update rate for the mixing probability to be $\tilde{\eta }=\frac{\eta }{\sigma }$. Such normalization eliminates the dependency in σ in all update equations. Larger values of $\tilde{\Delta }$ yield more segregation, as well as shorter build-up time (see part I of S2 Appendix). Importantly, $\tilde{\Delta }$ can be increased either by increasing Δ or by decreasing σ, and both Δ and σ have reasonable interpretations in actual experiments. Δ is the distance between the elements on the feature axis. When A and B represent pure tones, Δ is analogous to the frequency separation between them. Indeed, increasing the frequency separation in experiments results in higher segregation tendency.

The width of the conditional likelihoods, σ, can be related to the input presentation rate. We may conceive of classes as being represented by activity of neuronal populations, where the effective width of the classes becomes smaller when the rate is faster. One possible mechanism to implement this is differential suppression, described by Fishman et al [11], where at high stimulus presentation rates, responses to non-preferred frequencies are suppressed more than responses to the preferred frequency, narrowing the width of the responding neuronal population.

**Fig 7:**
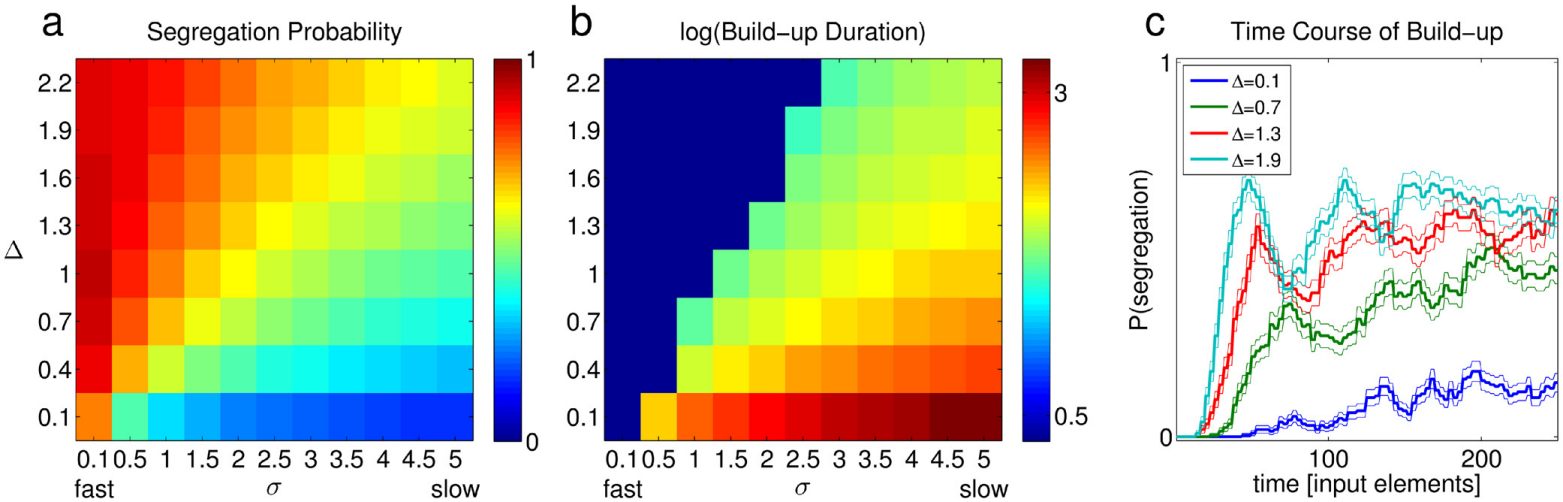
Results of ABA- simulations of the model under different values of Δ and σ. (a) The proportion of time spent in segregation. Small σ corresponds to fast sequences and vice versa, as indicated below the abscissa. (b) Duration of the initial integration percept in input elements count, shown in log values. Dark blue squares indicate conditions in which the initial percept was of segregation. In all simulations, the initial value for ***π*_2_** was 0.001, ***γ* = 0.1**, $\tilde{\eta }$ **=** **0.05**, and the shape of the conditional likelihood was generalized normal with **β = 0.5** and a variance of 1. The mixture was initialized with two classes centered at A and B, and ***π*_2_** was initially 0.001. Simulations were deterministic. On-going switching occurred in all simulations. (c) The time course of build-up, quantified as the probability of segregation among 100 stochastic simulations of each of Δ, for σ = 4. The thin line above and below each trace mark the standard error of the mean.

In Fig 7 we show the fraction of time spent in segregation (segregation probability) and the duration of the initial integration percept (build-up duration) as a function of Δ and σ, with $\tilde{\eta }$ kept constant. To get a better idea of the nominal segregation probability under each set of parameters, the simulations of Fig 7a and 7b did not use stochastic classification, i.e. each input was classified to class k* = *argmax*_*k*_ {*p*(*c* = *k*|*x*)}, and the resulting behavior was periodic switching as in Fig 4e. Stochastic simulations with the same parameters yielded a noisier picture with the same trends. It should be mentioned that since the quantities shown in Fig 7a and 7b only depend on the ratio between Δ and σ, namely $\tilde{\Delta }$, the same information could have been displayed in one dimension; the 2-d display conveys better the similarity of the results to the experimentally established dependency of these quantities in frequency separation and presentation rate.

In Fig 7b we fixed *π*_2_ to a constant value (0.001) at the beginning of each simulation, so that the initial integration durations could be compared across different values of Δ and σ. Fig 7c shows the time course of build-up, averaged over 100 stochastic simulations of each Δ, for σ = 4. For simulations with intermediate $\tilde{\Delta }$, where the steady state percept is ambiguous, the algorithm produces a default integration percept, followed by a build-up of segregation tendency, in accordance with experiments [4]. In agreement with the finding of Deike et al [5], Fig 7b shows immediate segregation for large $\tilde{\Delta }$ (dark blue squares), whereas small values of $\tilde{\Delta }$ yield extremely long initial integration percept, consistent with almost constant integration percept.

## Discussion

This paper presents a conceptual model of streaming that is based on the notion of evidence accumulation, together with a concrete implementation of this notion by a classification algorithm in which perceptual switching is achieved by the gradual update of the mixture probabilities. The idea underlying this model, that an accumulation process can generate positive correlation between subsequent phase durations, has been previously identified in the context of adaptation by Walker [25].

Indeed, our evidence accumulation process has superficial similarities with adaptation-based competition models. One class of these models, commonly used to describe bistable perception, features reciprocal inhibition: inhibition exerted by the dominant percept on the competing percept gradually becomes weaker due to a slow process that decreases the activity of the dominant percept (e.g., by spike frequency adaptation) or the connectivity between the two percepts (e.g., by synaptic depression) [19]. In the classification algorithm presented here, when an input close to *μ*_1_ arrives, *π*_2_ decreases, while when an input close to *μ*_2_ arrives, *π*_1_ decreases. In principle, this can be regarded as a manifestation of reciprocal inhibition between the classes; over time, the net change in *π*_2_, which favors the opposite percept, may seem analogous to the weakening of this reciprocal inhibition.

However, there is a conceptual difference between reciprocal inhibition models and the classification model: in the former, each population represents a percept, and the system operates in a regime where only one population is active at each time point, instantiating the bistable perception. In contrast, in the classification algorithm studied here, each class represents a stream; during integration all input elements are assigned to a single class, and during segregation input elements are assigned to two classes. The correlate of inhibition in our algorithm is thus not meant to generate exclusive activity of one class at a time, but rather to control the classification process.

The algorithm we propose accounts for many experimental constraints: positive correlation between subsequent phase durations is obtained, as well as the known dependency of segregation tendency and build-up on frequency separation and presentation rate. Still, the algorithm is presented here in a minimal form, providing a proof of concept rather than an accurate replication of experimental results. While even in this form it captures essential aspects of streaming, the implementation ignores important facets of real-world auditory stimuli. For example, there is no explicit representation of presentation rates, jittered sequences [26], or silent periods. Furthermore, we do not provide a general method to derive the representation of multidimensional inputs in the feature space. In contrast to pure tones that can be represented by a 1-dimensional feature space (frequency axis), general sounds have to be represented in a feature space with more than one dimension. Nevertheless, as previously mentioned, once a representation is established, the classification scheme can accommodate multidimensional feature spaces.

Previous studies had shown that inputs that arrive simultaneously to the scene are perceived as belonging to the same stream of sound [14]. In the model, synchronous elements are treated as a single element by default, since they would be considered as parts of a single complex sound rather than as separate entities. In this respect, the issue of synchronous elements would be treated in the model the same way a complex sound would.

To the best of our knowledge, the classification framework presented in this paper has not been suggested previously for streaming, although some previous studies are close in spirit. Martí and Rinzel [27] suggested a mechanism for formation of categories based on input statistics, maintained as bumps of activity on a line attractor; using this scheme, they demonstrated an analog for the build-up of segregation. Incoming inputs are not explicitly classified to one of the bumps, but since their statistics generated the bumps, it is possible to conceive of a simple mechanism to do that. Their model, however, does not show bistability for alternating stimuli. Another model that involves classification is the CHAINS model by Mill et al. [17], mentioned earlier; it is, however, tailored to periodic sound sequences, and therefore requires modifications to be applicable to general auditory sequences, that are not periodic by nature (e.g., speech). In contrast, our classification model assumes that streams are defined based on their statistical properties, hence it does not require periodicity, while still producing on-going switching in case of alternating sequences. Elhilali et al [28] also suggested a model for parsing acoustical scenes into streams based on predictions of future sounds. Their model is extremely general and provides a biologically plausible framework for segregation of complex signals, but it requires extensions to account for attributes of streaming such as bistability and gradual build-up.

Micheyl et al. suggest [12] that the size of the response to B in a neuron tuned to A determines perception—above a fixed threshold, the percept is integration, and below it, the percept is segregation. The classification algorithm we suggest seems similar in spirit to this decision rule, since the decision made by the algorithm for the ABA- sequence depends solely on the classification of B. Still, a neuronal implementation of our suggested algorithm would require additional features that are most likely not captured by the data collected by Micheyl et al. to test their decision rule. For example, updating class center may be modeled as plasticity of receptive fields; mixing probabilities may be represented in the form of synaptic strength (allowing a wide range of values for them, as required for probabilities), with synaptic plasticity representing their update.

While the framework and the model have been presented in the context of streaming, the classification algorithm has a substantially wider applicability. First, the classification algorithm yields a general mechanism for scene analysis. Dividing any dynamic scene into classes depends on the ability to identify the classes, simultaneous with the ability to assign new elements to them. This naturally calls for a classification algorithm.

Second, the proposed classification algorithm implements the default integration percept, as found in experiments. In the absence of sufficient information about the scene, for example when encountering a new auditory environment, the sensible solution is to classify all inputs to one default class, and to slowly reorganize perception as information accumulates. In this sense, the current model can be considered as a formal implementation of Bregman’s proposal that during an initial integration phase, the auditory system gradually accumulates evidence in favor of segregation into streams [29]. This occurs since the model initially tends to classify incoming inputs to “strong” existing classes (having a high mixing probability), while maintaining a mechanism of evidence accumulation. Indeed, the experimental finding of default integration and gradual build-up may be valid only when the steady state percept is ambiguous [5], as is the case for simulations with intermediate $\tilde{\Delta }$. The model also reproduces experimental results for extreme values of $\tilde{\Delta }$ (Fig 7b), where the gradual build-up of segregation does not hold; perception of such sequences can be interpreted as the analysis of a scene that is distinctly organized into one stream (for very small $\tilde{\Delta }$) or two separate streams of sound (for very large $\tilde{\Delta }$).

Third, the competition between classifying inputs solely to existing classes and classifying to a newly formed class situates scene analysis as a competition between more or less complex representations, as measured by the number of different classes (or by the entropy of the distribution over classes). This is a well-accepted trade-off in decision-making, for instance in the reinforcement learning literature where it is formulated as the “exploration-exploitation tradeoff” [30]. Exploitation, i.e. maximizing performance using existing knowledge only, would correspond to classification to active classes only; exploration of the sensory environment would correspond to classification to a newly formed class.

Partitioning the environment into streams of sound is arguably one of the most important tasks of auditory scene analysis. The different types of interpretations reported over time in response to a repeating stimulus indicate that perception does not necessarily converge to a single partitioning of the incoming sounds into streams. The online classification perspective suggested here is a dynamic process that provides a natural framework for dealing with complex scenes, captures the qualitative difference between interpretations of the same stimulus, and naturally accounts for the bistability in the perception of alternating sequences.

## Methods

### Existing Data Set (Data Set I [22])

We report data from two experiments. In the first experiment, 8 subjects were presented with 4-minutes long sequences of repeating ABA- triplets. They reported their perception of each stimulus by continuously pressing or releasing a mouse button, to indicate segregation or integration respectively. The original aim of this experiment was to explore the relationship between auditory and visual bistability, therefore trials of auditory stimulus, visual stimulus or combined auditory and visual stimulus were presented in random order. We only analyzed the pure auditory trials (6 per subject). The second experiment replicated the first with a different visual task, thus adding to our analysis 8 more subjects, of which 6 subjects participated also in the first experiment (in consequences, there are 10 subjects in total). Still, we treat the data sets as coming from 16 different subjects (the two experiments were performed at different times; analyzing the data as coming from 10 subjects did not modify the conclusions). In all 96 trials from 16 subjects, tone A was 587 Hz, and tone B was 440 Hz. In the first experiment, tone duration was 120 ms, and tones were presented over headphones. In the second experiment, tone duration was 240 ms, A tones were presented from loudspeakers to the left of the subject and B tones from loudspeakers to the right of the subject. Onset to onset time was always equal to tone duration.

### Data Set II

#### Ethics statement

All experimental protocols received approval of the Ethics Committee of the Hebrew University in Jerusalem. Human participants provided a written consent to participate in experiments.

#### Participants

21 subjects (aged 24–52, median age 30, 9 females) were recruited from a student population known to the experimenter. All subjects reported normal hearing.

#### Apparatus

The psychophysical measurements were performed in a quiet room. The stimuli were digitally synthesized in Matlab (The Mathworks Inc., Natick, MA, USA), fed into a Saffire 6 USB hardware interface (Focusrite Audio Engineering Ltd., UK), and presented binaurally through HDA 200 Audiometric headphones (Sennheiser) at about 63 dB SPL.

#### Stimuli and Procedure

Participants were presented with 6 repetitions of 4-minutes long sequences of repeating ABA- triplets. Integration was defined to the subjects as hearing the sequence as one alternating melody, whereas segregation was defined as perceiving the high tones separately from the low tones. In uncertain situations, subjects were instructed to choose the better fitting option. Subjects reported changes in their perception by pressing a keyboard button at the beginning of each phase: “1” for integration and “2” for segregation. The timing of the key press was considered as the beginning of the next phase as well as the end of the previous phase. The interval preceding the first key press was disregarded. Other studies use a different reporting scheme where the subjects press the key as long as perception endures (pressing for segregation and releasing for integration, as done in the study of Data Set I, or alternatively pressing one key throughout integration, and another throughout segregation). The approach we employed assumes that integration and segregation are the only possible percepts of the sequence, an assumption that may be in conflict with reports of a “third” response type [3]. We reasoned that reporting the transition points was easier for the subject than reporting the entire phase duration, and therefore more accurate, since it requires noticing changes, rather that making a decision at each and every time point. The effect of “gluing” the unknown phases to integration and segregation was mitigated by the instructions to choose the better fitting option, forcing the subjects to categorize the unknown phases to one of the defined categories. Tone A was roved (frequencies: 440, 494, 554 Hz), to reduce the monotonicity of the experiment. Tone B was always 5 semitones above tone A. Onset to onset time was 110 ms, and tone duration was 50 ms; our experience suggests that it is easier to explain segregation to naïve subjects when the tones are shorter than the onset to onset time.

### Correlation Scatter (Fig 1)

When calculating the correlation coefficients between the durations of successive perceptual phases, artifactual correlations of two types can arise:

1. Within subject: switching rate from integration to segregation and switching rate from segregation to integration may be different for the same subject, resulting in a negative correlation when calculating the correlation for both switching types together. To avoid such correlation, we displayed the scatter plots and analyzed different switching types separately (I→S transition separately from S→I).
2. Between subjects: some subjects are fast switchers and some are slow, and this yields positive correlation when calculating the correlations for all subjects together. To avoid such correlation, we normalize by the average duration for each perceptual phase type in each trial (and therefore subject) separately. Such normalization concentrates all points of a trial around (1,1).

We exclude the first and last phases from the analysis. We did not use any other exclusion criterion for the results reported in the main text. In Part A in S1 Appendix, we compare these results with the results of the same analyses when trials with less than 10 perceptual switches were excluded from the analysis. The motivation for this criterion is that for such trials the estimation of the mean duration is poor, affecting their contribution to the overall correlation (Fig 1), and the inter-trials correlation is poorly estimated, affecting the results reported in Fig 2.

Throughout the paper, we tested correlations for significance at p<0.01. Standard significance tests on correlation coefficients are usually stated for normally distributed variables only. However, the same tests are asymptotically correct for any distribution that would fulfill the conditions of the central limit theorem, so they are approximately correct in our case, due to the large number of data points.

### Linear mixed models for testing correlations (Fig 2)

The correlation values in the histogram of Fig 2 were calculated separately for each trial and transition type, so normalization was not required. Still, for testing the average correlation, we had to allow for within-subject tendency of the correlation coefficients to cluster. In consequence, we modeled the correlations as *ρ*_*ij*_ = *μ* + *β*_j_ + *ε*_*ij*_, where ρ_ij_ is the correlation in the i^th^ trial of the j^th^ subject, μ is the overall mean (to be tested against 0), β_j_ are random subject effects, and ε_ij_ is the error. The model was fitted in R [31] using the routine *lmer* (package *lme4* [23]) and tested using the package *lmerTest* [32].

#### Generalized Normal Distribution

To allow freedom in the shape of the conditional likelihoods *p*(*x*|*c* = *k*), we used the generalized normal distribution, where a shape parameter β determines the heaviness of the tail:

$$p\left(x|c=k\right)=\frac{\beta }{2\alpha \Gamma \left(\frac{1}{\beta }\right)}e^{-{\left|\left(x-\mu \right)/\alpha \right|}^{\beta }}$$

Γ(·)denotes the Gamma function.

Specifically, β = 2 gives a normal distribution. β was determined once, prior to the simulation, uniformly for all classes. The switching behavior of the model was observed for a variety of values for β; in the simulations above we chose distributions with a heavier tail than normal, i.e. β<2.

## Supporting Information

S1 Appendix (DOCX)

S1 FigRelative addition to the mixing probability *π*_1_, defined as $\frac{\pi_{1}\left(t+1\right)-\pi_{1}\left(t\right)}{\pi_{1}\left(t\right)}$, as a function of the input element, calculated for the case where two classes exist, for (a) the streaming algorithm, and (b) the speech categorization algorithm.Different colors represent different values of ***μ*_1_ –*μ*_2_**, as indicated by the vertical lines. The abscissa is aligned around the middle between ***μ*_1_** and ***μ*_2_**. In both panels, ***π*_1_ = 0.4**.(EPS)

S1 Dataset Data collected for Data Set II. The structure “trial” is of size 21 X 6 (subject X trial). In each entry, the vector “data” contains switch times in seconds, and the corresponding vector “percept” contains the percept type to which there was a switch (1 = integration, 2 = segregation). The field “freqL” is the frequency of tone A in Hz.
(MAT)
