# Supplementary material for: Auditory Streaming as an Online Classification Process with Evidence Accumulation
Source: PLoS One. 2015 Dec 15;10(12):e0144788. doi: 10.1371/journal.pone.0144788 (PMC4699212; doi:10.1371/journal.pone.0144788)
Supplement: S1 Appendix — (DOCX) [file pone.0144788.s001.docx]

**S1 Appendix**

**Part A: Correlation Analysis with and without Excluding Trials**

**Correlation across Phase Durations from All Subjects (Fig 1 in the main text)**

The following tables show the correlation analysis of Fig 1 of the main text in two methods: without any exclusion criterion, and excluding trials that have less than 10 perceptual switches. The motivation for this criterion is that for such trials the estimation of the mean duration is poor, affecting their contribution to the overall correlation. In both tables, each line shows one transition type in one data set.

Columns show the number of phase couples used for calculation n, the correlation coefficient ρ and the p value respectively. The results from table a are also elaborated in the text and in Fig 1 of the main text, and are summarized here for comparison with table b.

a) Using all trials

|  | n | ρ | p |
| --- | --- | --- | --- |
| **Data Set I** |  |  |  |
| lag1 I->S | 1563 | 0.267 | 7.64E-27 |
| lag1 S->I | 1609 | 0.267 | 1.26E-27 |
| lag2 I->I | 1515 | 0.049 | 0.058 |
| lag2 S->S | 1563 | 0.051 | 0.045 |
| lag3 I->S | 1469 | 0.015 | 0.557 |
| lag3 S->I | 1515 | -0.053 | 0.038 |
| **Data Set II** |  |  |  |
| lag1 I->S | 1469 | 0.179 | 5.16E-12 |
| lag1 S->I | 1523 | 0.091 | 3.64E-04 |
| lag2 I->I | 1410 | 0.013 | 0.635 |
| lag2 S->S | 1460 | 0.049 | 0.064 |
| lag3 I->S | 1348 | 0.008 | 0.777 |
| lag3 S->I | 1401 | -0.025 | 0.357 |

b) Excluding trials that have less than 10 perceptual switches

|  | n | ρ | p |
| --- | --- | --- | --- |
| **Data Set I** |  |  |  |
| lag1 I->S | 1557 | 0.268 | 5.59E-27 |
| lag1 S->I | 1601 | 0.267 | 1.26E-27 |
| lag2 I->I | 1510 | 0.050 | 0.053 |
| lag2 S->S | 1557 | 0.052 | 0.040 |
| lag3 I->S | 1466 | 0.016 | 0.549 |
| lag3 S->I | 1510 | -0.054 | 0.035 |
| **Data Set II** |  |  |  |
| lag1 I->S | 1415 | 0.182 | 4.96E-12 |
| lag1 S->I | 1459 | 0.090 | 6.18E-04 |
| lag2 I->I | 1366 | 0.022 | 0.423 |
| lag2 S->S | 1410 | 0.058 | 0.029 |
| lag3 I->S | 1317 | 0.038 | 0.174 |
| lag3 S->I | 1361 | -0.027 | 0.317 |

Correlation values are therefore similar with and without exclusion.

**Linear Mixed Effect Analysis of the Inter-Trial Correlation (Fig 2 in the main text)**

The following tables show the linear mixed effect analysis of the inter-trial correlation with subject as a random factor, in two methods: without any exclusion criterion, and excluding trials that have less than 10 perceptual switches. The motivation for this criterion is that for such trials the inter-trials correlation is poorly estimated. In both tables, each line shows one transition type in one data set.

Columns show the number of trials used for calculation, the mean correlation, the standard deviation of the correlation (std), t, df and p value of the fixed effect, and χ, df and p value of the random subject effect respectively. Most of the results from table a are elaborated in the text and in Fig 2 of the main text, and are summarized here for comparison with table b.

a) Using all trials for which it is possible to calculate the correlation

|  |  | **fixed effect** | | | | | **random effect** | | |
| --- | --- | --- | --- | --- | --- | --- | --- | --- | --- |
|  | # trials | mean | std | df | t | p | χ | df | p |
| **Data Set I** |  |  |  |  |  |  |  |  |  |
| lag1 I->S | 92 | 0.225 | 0.376 | 15.168 | 5.31 | 8.46E-05 | 0.23 | 1 | 0.6 |
| lag1 S->I | 92 | 0.249 | 0.312 | 15.352 | 6.69 | 6.41E-06 | 0.77 | 1 | 0.4 |
| lag2 I->I | 92 | -0.027 | 0.313 | 15.477 | -0.64 | 0.531 | 2.83 | 1 | 0.09 |
| lag2 S->S | 92 | 0.025 | 0.303 | 91 | 0.80 | 0.427 | 0 | 1 | 1 |
| lag3 I->S | 91 | 0.016 | 0.340 | 14.39 | 0.34 | 0.738 | 2.98 | 1 | 0.08 |
| lag3 S->I | 92 | -0.061 | 0.363 | 91 | -1.60 | 0.112 | 2.84E-14 | 1 | 1 |
| **Data Set II** |  |  |  |  |  |  |  |  |  |
| lag1 I->S | 109 | 0.176 | 0.398 | 10.763 | 3.78 | 0.003 | 1.08 | 1 | 0.3 |
| lag1 S->I | 112 | 0.131 | 0.374 | 16.072 | 2.58 | 0.020 | 6.39 | 1 | 0.01 |
| lag2 I->I | 106 | -0.075 | 0.348 | 105 | -2.23 | 0.028 | 0 | 1 | 1 |
| lag2 S->S | 108 | -0.022 | 0.364 | 13.709 | -0.61 | 0.554 | 0.03 | 1 | 0.9 |
| lag3 I->S | 103 | 0.039 | 0.385 | 102 | 1.03 | 0.304 | 0 | 1 | 1 |
| lag3 S->I | 105 | -0.067 | 0.434 | 16.681 | -1.58 | 0.133 | 1.74E-03 | 1 | 1 |

b) Excluding trials that have less than 10 perceptual switches

|  |  | **fixed effect** | | | | | **random effect** | | |
| --- | --- | --- | --- | --- | --- | --- | --- | --- | --- |
|  | # trials | mean | std | df | t | p | χ | df | p |
| **Data Set I** |  |  |  |  |  |  |  |  |  |
| lag1 I->S | 91 | 0.238 | 0.356 | 90 | 6.38 | 7.57E-09 | -2.84E-14 | 1 | 1 |
| lag1 S->I | 91 | 0.245 | 0.312 | 15.00 | 6.33 | 1.35E-05 | 1.21 | 1 | 0.3 |
| lag2 I->I | 91 | -0.022 | 0.309 | 15.14 | -0.55 | 0.594 | 1.77 | 1 | 0.2 |
| lag2 S->S | 91 | 0.035 | 0.290 | 90 | 1.14 | 0.256 | -4.26E-14 | 1 | 1 |
| lag3 I->S | 91 | 0.016 | 0.340 | 14.39 | 0.34 | 0.738 | 2.98 | 1 | 0.08 |
| lag3 S->I | 91 | -0.062 | 0.364 | 90 | -1.63 | 0.108 | -4.26E-14 | 1 | 1 |
| **Data Set II** |  |  |  |  |  |  |  |  |  |
| lag1 I->S | 98 | 0.193 | 0.354 | 15.72 | 4.54 | 3.49E-04 | 1.32 | 1 | 0.3 |
| lag1 S->I | 98 | 0.119 | 0.328 | 16.41 | 2.80 | 0.013 | 2.97 | 1 | 0.08 |
| lag2 I->I | 98 | -0.038 | 0.321 | 97 | -1.17 | 0.246 | 0 | 1 | 1 |
| lag2 S->S | 98 | -0.001 | 0.329 | 97 | -0.04 | 0.972 | 0 | 1 | 1 |
| lag3 I->S | 98 | 0.053 | 0.377 | 97 | 1.39 | 0.166 | 0 | 1 | 1 |
| lag3 S->I | 98 | -0.066 | 0.395 | 16.44 | -1.60 | 0.128 | 0.03 | 1 | 0.9 |

The deviation from zero of the distribution of inter-trial correlation is therefore similar with and without exclusion.

Note that in Data Set II, a large proportion of the trials were excluded when using the 10 switches exclusion criterion (28/126, 22.2%) compared to Data Set I (5/96, 5.2%). Even without exclusion (table a), a large fraction of trials have not been used due to their number of switches being too small to calculate the correlation, compared to Data Set I. In a separate, smaller data set collected by Pressnitzer and Hupé [1], where the same stimuli and apparatus as in the first half of Data Set I were used, the same exclusion criterion yielded 6/23 excluded trials (26.1%). It is therefore reasonable to assume that inter-subject variability allows such disparity. Interestingly, we found in Data Set II (collected by us) that 12/28 of the excluded trials came from two subjects with extreme values for phase durations (and therefore a very small number of switches).

**Part B: Aspects of the Classification Model**

**Effect of** $\tilde{\boldsymbol{\Delta}}$ **on Segregation Tendency**

In the deterministic classification case, the decision boundary between integration and segregation in the $\pi_{2}-\mu_{1}$ space is defined by the line $\pi_{2}P\left( B|c=2 \right)=\left( 1-\pi_{2} \right)P\left( B|c=1 \right)$. Assuming $\mu_{2}=B$ we get

$$\pi_{2}=\frac{f\left( B-\mu_{1} \right)}{1+f\left( B-\mu_{1} \right)}$$

where $P\left( B|c=1 \right)\equiv\alpha f\left( B-\mu_{1} \right)$, such that $f\left( 0 \right)=1$.

For a given value of $\mu_{1}$, $\pi_{2}$ values above this value yield segregation decision, while values below yield integration decision. Larger $\tilde{\Delta}$ yields smaller $f\left( B-\mu_{1} \right)$, shifting the decision boundary towards smaller values of $\pi_{2}$, and increasing the portion of the plane where segregation is decided. This is of course not a proof for larger segregation tendency in the case of larger $\tilde{\Delta}$, but at the very least it shows that this trend is reasonable, as clearly shown in simulations (Fig 7).

**Relation to Expectation-Maximization Algorithm**

Nowlan [2] has developed an online Expectation-Maximization (EM) algorithm, which was later justified by Neal et al. [3] (their equation 10). We describe the similarity between the streaming algorithm and this EM algorithm, and explain why despite this similarity, the EM algorithm does not feature competition between processes. The update procedure of the online EM algorithm is purely online, i.e. it only uses the current input element to perform calculations of the parameters update. Applying this algorithm to a Gaussian mixture (i.e., we choose β=2 for concreteness), we get the following set of update rules for ${\pi_{k}}^{t},{\mu_{k}}^{t}$ given their previous values ${\pi_{k}}^{t-1},{\mu_{k}}^{t-1}$ when an input element $x$ arrives (There is also an update equation for ${\sigma_{k}^{2}}^{t}$, which we disregard here since it is not updated in our model):

$${\pi_{k}}^{t}={\pi_{k}}^{t-1}\left[ 1+\left( 1-\gamma\right)\frac{\frac{p^{t}\left( x|c=k \right)}{p^{t}\left( x \right)}-1}{1-\left( 1-\gamma\right)\frac{p^{t}\left( x|c=k \right)}{p^{t}\left( x \right)}} \right]$$

$${\mu_{k}}^{t}={\mu_{k}}^{t-1}+\left( 1-\gamma\right)\frac{p^{t}\left( x|c=k \right)}{p^{t}\left( x \right)}\left( x-{\mu_{k}}^{t-1} \right)$$

where $0<\gamma<1$ is a parameter. This calculation is valid after enough steps, when the sum of the sufficient statistics used for normalization in estimating $\pi_{k}$ reaches a steady state value.

For $\pi_{k}$ to increase due to this update, it is required that $p^{t}\left( x|c=k \right)>p^{t}\left( x \right)>{\left( 1-\gamma\right)p}^{t}\left( x|c=k \right)$. The values of these likelihoods are calculated after the update, so the requirement is somewhat circular, but it is still reasonable to assume that classes that satisfy $p^{t}\left( x|c=k \right)>p^{t}\left( x \right)$ after the update also yielded the highest (or at least among the higher) conditional likelihoods prior to the update. As for the $p^{t}\left( x \right)>{\left( 1-\gamma\right)p}^{t}\left( x|c=k \right)$ requirement, it seems to limit the ratio between the conditional likelihood and the total likelihood to values not too big; still, if we assume a value of $\gamma$ close to 1, it is easy to satisfy. The bottom line is that the weight update scheme favors classes with high conditional likelihoods, similar to the update scheme we use in our proposed model.

$\mu_{k}$ values approach the input x per its arrival, in an amount proportional to the distance from it, similarly to the update in our model. There are two main differences between this update and the update we use (equation (1) in the main text). First, here all class centroids are updated, whereas in our model only one selected class is updated. In fact, our model can also be presented with a “soft” update scheme, where all centroids are updated based on the same formula, and yet preserve its qualitative behavior (internal simulations). The second difference lies in the coefficient of the step size; here it is $\frac{p\left( x|c=k \right)}{p\left( x \right)}$, whereas in our model it is $p\left( c=k|x \right)$ which is equal to $\frac{p\left( x|c=k \right)\pi_{k}}{p\left( x \right)}$. Obviously, the EM update scheme favors classes with high conditional likelihood, and not classes with high a-posteriori probability as in our model.

There is therefore no mixture of criteria in the EM algorithm; alternating inputs will not yield competition and switching between the mixture states. The class chosen for classification is always the one that is strengthened in the sense of the mixing probability increase. Arrival of a new kind of inputs will terminate in either their classification to a brand new class, of their classification to an existing class. Specifically, for alternating stimulus there will be convergence to either integration or segregation, with no switching. In the latter case, segregation will begin immediately with the first presentation of B. It is therefore clear that there is no competition between processes, and no evidence accumulation.

**Relation to Speech-Learning Algorithm**

McMurray et al. [4] suggested an algorithm for learning vowel categories from speech, based on a Gaussian mixture distribution. We describe the similarity between the streaming algorithm and this speech-learning algorithm, and explain why despite this similarity, the speech-learning algorithm does not feature competition between processes. The update procedure for ${\pi_{k}}^{t},{\mu_{k}}^{t}$ given their previous values $\pi_{k},\mu_{k}$ when an input element $x$ arrives are (again, there is also an update equation for $\sigma_{k}$, which we disregard here since it is not updated in our model):

$$\pi_{k}\leftarrow\pi_{k}\left[ 1+\frac{\eta}{1+\eta}\left( \frac{p\left( x|c=k \right)}{p\left( x \right)}-1 \right) \right]$$

$$\mu_{k}\leftarrow\mu_{k}+\gamma p\left( c=k|x \right)\frac{\left( x-\mu_{k} \right)}{\sigma_{k}^{2}}$$

The centroid update accentuates classes that yield high a-posteriori probability, similar to what we suggest in our algorithm. In contrast to the centroid update, and in similarity to our algorithm, the mixing probabilities are updated accentuating the class that yields maximum conditional likelihood, presumably forming a competing process; however, no competition is formed here. The reason is that the weights given to incoming elements in the mixing probability update do not allow this competition: inputs are not weighted by their conditional likelihood, rather by their distance from the boundary between classes, such that the contribution of far inputs to the mixing probability of their closest class is similar to that of near inputs. S1 Fig shows the relative addition to $\pi_{1}$, defined as $\frac{\pi_{1}\left( t+1 \right)-\pi_{1}\left( t \right)}{\pi_{1}\left( t \right)}$, for each input value, in the case where two classes exist. Both in our algorithm and in McMurray et al.’s algorithm, when the input $x$ is closer to $\mu_{1}$, $\pi_{1}$ increases (positive addition), and otherwise it decreases. The difference between the algorithms is in the monotonicity of the relative addition function. To demonstrate the consequences of this difference, assume that $\mu_{1}$ is centered at A and $\mu_{2}$ at B, and that both elements are classified into class 1. $\mu_{1}$ approaches the middle between A and B, while $\mu_{2}$ remains at B (in S1 Fig this amounts to taking smaller $\mu_{1}-\mu_{2}$ value and considering A to the right of $\mu_{1}$). At this point, to get competition, $\pi_{1}$ needs to decrease, such that class 2 can try and compete class 1. Indeed, in the streaming algorithm (S1 Fig a), the contribution of A to $\pi_{1}$ becomes smaller, while the negative contribution of B to $\pi_{1}$ remains significant, and all in all $\pi_{1}$ decreases. This happens since the addition size decreases to zero as $x\to\infty$; far inputs do not provide evidence for the relevance of the class, thus they do not contribute to its mixing probability. In contrast, for the speech-learning algorithm (S1 Fig b), the relative addition to $\pi_{1}$ is monotonically increasing, such that far inputs on the right half of the axis contribute more than inputs in the vicinity of $\mu_{1}$. As $\mu_{1}$ approaches the middle between A and B, the contribution of A to $\pi_{1}$ is comparable in size with (or even larger than) the negative contribution of B to $\pi_{1}$, and it is not guaranteed that $\pi_{1}$ will decrease. The qualitative difference between the algorithms in the scenario explained here becomes more and more prominent as Δ increases; for small values of Δ, competition is not guaranteed also for our algorithm. This is in line with properties of streaming, known to yield constant integration for small frequency separation between A and B.

**References**

1. Pressnitzer D, Hupé JM. Temporal dynamics of auditory and visual bistability reveal common principles of perceptual organization. Curr Biol. 2006;16: 1351–1357. doi:10.1016/j.cub.2006.05.054

2. Nowlan SJ. Soft Competitive Adaptation: Neural Network Learning Algorithms Based on Fitting Statistical Mixtures. Carnegie Mellon University. 1991.

3. Neal RM, Hinton GE. A view of the EM algorithm that justifies incremental, sparse, and other variants. Learning in graphical models. Springer; 1998. pp. 355–368. Available: http://link.springer.com/chapter/10.1007/978-94-011-5014-9_12

4. McMurray B, Aslin RN, Toscano JC. Statistical learning of phonetic categories: insights from a computational approach. Dev Sci. 2009;12: 369–378. doi:10.1111/j.1467-7687.2009.00822.x
